# An Anisotropic 4D Filtering Approach to Recover Brain Activation From Paradigm-Free Functional MRI Data

**DOI:** 10.3389/fnimg.2022.815423

**Published:** 2022-04-01

**Authors:** Isa Costantini, Rachid Deriche, Samuel Deslauriers-Gauthier

**Affiliations:** Inria, Université Côte d'Azur, Valbonne, France

**Keywords:** BOLD deconvolution, functional MRI, image regularization, paradigm free, resting-state, anisotropic regularization

## Abstract

**Context:**

Functional Magnetic Resonance Imaging (fMRI) is a non-invasive imaging technique that provides an indirect view into brain activity *via* the blood oxygen level dependent (BOLD) response. In particular, resting-state fMRI poses challenges to the recovery of brain activity without prior knowledge on the experimental paradigm, as it is the case for task fMRI. Conventional methods to infer brain activity from the fMRI signals, for example, the general linear model (GLM), require the knowledge of the experimental paradigm to define regressors and estimate the contribution of each voxel's time course to the task. To overcome this limitation, approaches to deconvolve the BOLD response and recover the underlying neural activations without a priori information on the task have been proposed. State-of-the-art techniques, and in particular the total activation (TA), formulate the deconvolution as an optimization problem with decoupled spatial and temporal regularization and an optimization strategy that alternates between the constraints.

**Approach:**

In this work, we propose a paradigm-free regularization algorithm named Anisotropic 4D-fMRI (A4D-fMRI) that is applied on the 4D fMRI image, acting simultaneously in the 3D space and 1D time dimensions. Based on the idea that large image variations should be preserved as they occur during brain activations, whereas small variations considered as noise should be removed, the A4D-fMRI applies an anisotropic regularization, thus recovering the location and the duration of brain activations.

**Results:**

Using the experimental paradigm as ground truth, the A4D-fMRI is validated on synthetic and real task-fMRI data from 51 subjects, and its performance is compared to the TA. Results show higher correlations of the recovered time courses with the ground truth compared to the TA and lower computational times. In addition, we show that the A4D-fMRI recovers activity that agrees with the GLM, without requiring or using any knowledge of the experimental paradigm.

## 1. Introduction

Functional MRI (fMRI) is a non-invasive imaging technique that indirectly probes brain function by providing a measure of the metabolic activity consequent to an increased neural activation. Two experimental setups are commonly used to acquire fMRI data, task-fMRI, and resting-state fMRI (rs-fMRI). In the former, the subject is asked to follow an experimental paradigm, whereas in the latter the subject is asked to rest in the scanner and not do or think of anything in particular. Standard approaches for the analyses of task-fMRI data are based on the well-known general linear model (GLM) adapted by Friston and colleagues in 1998 in the context of fMRI data analyses (Friston et al., 1998). This approach requires prior knowledge of the task parameters and timing of events, as well as assumptions about the neural and hemodynamic responses. Therefore, the GLM can be used only for task-fMRI experiments, where the expected stimulus response is given by experimental paradigm. In contrast to task-fMRI, where the focus is on the response to a specific stimulus, rs-fMRI provides insight on brain function in the absence of stimuli. It also allows us to map the brain activity of patients whose condition does not allow them to perform tasks or follow an experimental paradigm. Furthermore, there are also brain activations that cannot be modeled and expected, such as seizures in epileptic patients (Karahanoğlu et al., 2013). For these data, which represent unpredictable brain activity, the GLM approach is not suitable (Gusnard and Raichle, 2001).

Data-driven methods have been proposed to analyze images obtained in resting-state, when no information about the occurrence of the activation is available. They include blind source separation approaches such as the independent component analysis (ICA) (McKeown et al., 1998; Beckmann and Smith, 2004; Calhoun and Adali, 2006), the principal component analysis (PCA) (Andersen et al., 1999; Baumgartner et al., 2000), the temporal clustering analysis (Liu et al., 2000; Morgan et al., 2008), and clustering methods (Cordes et al., 2002; Salvador et al., 2005; Golland et al., 2008; Lee et al., 2012). These methods are of interest if the aim is to group voxels showing the same spatial or temporal features but they cannot be used if the goal is identifying activations at the voxel level. Indeed, they do not consider including any hemodynamic effect. They are also limited by the necessity of choosing a priori the number of components or clusters and by their interpretation (Gaudes et al., 2011).

To overcome these limitations, deconvolution approaches have been developed to address the problem of studying and uncovering brain activations hidden within fMRI time series at the voxel level. fMRI deconvolution was introduced by Glover in Glover (1999), who investigated the performance of Wiener deconvolution for deblurring the fMRI response and reduce image distortions. This approach resulted in smooth recovered activation (Karahanoğlu et al., 2013) and required an independent measurement of the noise spectral density (Gitelman et al., 2003). Gitelman et al. (2003) developed an approach based on linear deconvolution and modeled the interplay between areas as effects of psycho-physiological interactions. In addition, dynamical filter methods, such as Kalman and Bayesian filtering, and local linearization filters have been developed and applied to fMRI (Riera et al., 2004; Friston et al., 2008; Havlicek et al., 2011). However, because these approaches are based on non-linear models in continuous time, they are limited by the high computational cost and convenient only for the analysis of localized regions of interests (ROIs) (Karahanoğlu et al., 2013). Other approaches make spatial and/or temporal assumptions on the underlying signals, thus adding priors in the optimization problems. In particular, sparse regularization on the recovered activation maps was exploited to force to zero the weights of regressors, which did not contribute to the activation (Flandin and Penny, 2007; Smith and Fahrmeir, 2007; Harrison et al., 2008). L1-norm regularization approaches have also been developed to exploit sparse temporal features of the hidden neural activation. This was done by means of the majorization–minimization of a cost function to find an optimal solution to the inverse problem (Hernandez-Garcia and Ulfarsson, 2011). Caballero Gaudes et al. developed a ridge-regression regularization by minimizing both the variance of the residuals and the power of the resulting estimate of the input signal representing the brain activity (Gaudes et al., 2011) and a sparse regression (Caballero Gaudes et al., 2013) by assuming short neuronal activations. Temporal regularized optimization problems based on wavelets were also explored (Khalidov et al., 2011). These methods exploit the temporal features of the hemodynamic response function (HRF) (Karahanoğlu et al., 2013) attempting to deconvolve it from the fMRI data, rather than using any information on the timing of the neural activations which translate on the blood oxygen level dependent (BOLD) response. Recently, by supposing the brain activates in constant blocks, Karahanoğlu et al. (2013), later revisited by Farouj et al. (2017), developed a deconvolution approach which involves both spatial and temporal regularization called total activation (TA). These approaches split the optimization problem into two decoupled spatial and temporal regularizations that increases the number of parameters to be set and requires the solver to alternate between the constraints. The temporal regularization of the latter was then improved by Costantini et al. (2018) by proposing a joint approach using the least angle regression (LARS) algorithm and the L-curve, which overcame the limitation of having to choose *a priori* the regularization parameter, and reduced the computation time. Recently, deconvolution algorithms have also incorporated a multivariate formulation to perform spatiotemporal deconvolution in 2D (Uruńuela et al., 2020, 2021). The first allows the estimation of blocks of activations coupled with a subsampling approach based on stability selection to avoid the choice of the regularization parameter. The latter enables a method that finds global fluctuations due to motion artifacts or physiological signals as well as neuronal activity. Also, Bolton et al. (2019) proposed a spatiotemporal deconvolution approach that includes structurally informed regularization.

Our approach is based on the idea that brain activations occur in spatially and temporally coherent patterns with sharp boundaries. These patterns are projected to fMRI data *via* the HRF and corrupted by physiological and motion artifacts. To recover the underlying brain activations, we propose a novel method based on partial differential equations (PDEs), named anisotropic 4D filtering fMRI (A4D-fMRI). The A4D-fMRI applies an anisotropic diffusion process whose diffusivity is steered by derivatives of the evolving image to smooth the fMRI image and simultaneously enhance important features such as spatial edges and temporal functional activations. Regularization methods have been enriched by the use of non-linear PDEs in several contexts for the last 30 years. First applied to physics and fluid mechanics, it has been shown that non-linear PDEs allow smoothing the data while preserving large global features, such as discontinuities of the signal (Tschumperle and Deriche, 2005), which can be found, for example, in image contours and corners (Tschumperlé and Deriche, 2007). This approach is based on the isotropic diffusion equation, i.e., heat flow, and has subsequently been extended to other theoretical contributions. Among them there are the anisotropic smoothing (Weickert, 1998; Sapiro, 2006) and the PDEs-based gradient descent used to solve energy functionals minimizations (Rudin et al., 1992; Chambolle and Lions, 1997; Charbonnier et al., 1997; Kimmel et al., 2000; Aubert and Kornprobst, 2006). The pioneering work that employed anisotropic diffusion PDEs for the restoration of noisy and blurred digital data was proposed by Perona and Malik (1990) overcoming the limitations associated with linear filtering approaches (Tschumperle and Deriche, 2002). To date, PDEs-based regularization algorithm has been applied to 2-D scalar images (Perona and Malik, 1990; Nielsen et al., 1997; Weickert, 1998; Aubert and Kornprobst, 2006) and vector-valued images (Tschumperle and Deriche, 2002).

The A4D-fMRI proposed in this work has been conceived for the geometrical regularization of 4D fMRI images (3D space × 1D time) based on PDEs. The A4D-fMRI acts concurrently in space and time, thus overcoming the limitation of previous deconvolution approaches that consider the two problems of spatial and temporal regularization as decoupled processes. In our method, the regularization flow is performed according to the time and to the local geometry of the image to evaluate the presence of an edge and its local strength.

The rest of the article is organized as follows. We first introduce the PDEs regularization theoretical framework, illustrate the mathematical problem, and how we solved it. Next, we validate the A4D-fMRI on phantom data and on task-fMRI data from 51 subjects using the experimental paradigm as ground truth and positively compare its performance to the TA approach. We also applied the A4D-fMRI approach to rs-fMRI data. Finally, the advantage of our approach of not requiring any knowledge of the experimental paradigm is shown by recovering activity that agrees with the GLM, which uses and need this *a priori* knowledge.

## 2. Theory

The fMRI BOLD signal is a combination of brain activation, the HRF, physiological artifacts, and noise. More specifically, fMRI data are modeled as a brain activation that is convolved with the HRF and corrupted by noise. So, starting from the corrupted fMRI images, the purpose of this work is to remove from this signal the noise and the hemodynamic effect and to keep only the underlying activation signal that is at its origin. To achieve this goal, in this paper we propose to solve this regularization problem using diffusion theory. Inspired by the physics of fluids, many authors assimilated the process of image regularization with the diffusion of chemical concentrations and proposed to apply the following diffusion PDE process (Weickert, 1998, 1999; Tschumperle and Deriche, 2002, 2005; Tschumperlé and Deriche, 2007),


(1) $$\frac{\partial \text{I}}{\partial \text{t}}=\text{div}(\text{D}\nabla \text{I})$$

where **I** is the input image, ∇ is the gradient operator, *t* is the time, div(·) is the divergence operator, and


(2) $$\text{D}=\lambda_{1}\text{u}{\text{u}}^{T}+\lambda_{2}\text{v}{\text{v}}^{\text{T}}$$

is the diffusion tensor of the image **I**, also called structure tensor (Förstner, 1986; Förstner and Gülch, 1987; Tschumperle and Deriche, 2005). The diffusion tensor **D** is a symmetric and positive definite matrix, and has λ_1_, λ_2_ as positive eigenvalues and **u, v** as corresponding orthogonal eigenvectors that drive the regularization process; the amount of diffusion in the directions **u** and **v** will be weighted by λ_1_ and λ_2_, respectively. PDEs smooth the image at each step with a notion of scale-space (Perona and Malik, 1990; Nielsen et al., 1997; Lindeberg, 2013). The scale-space technique involves generating coarser resolution images by convolving the original image with a Gaussian kernel. Therefore, starting from an original image, according to one parameter, i.e., the size of the smoothing kernel, a set of smoothed images are produced. At each iteration, the image is smoothed and fine-scale properties, such as noise in our case, are gradually suppressed. To apply the diffusion process to fMRI data, Equation (1) will need to be reformulated to handle 4D images. Let us define a scalar-valued image as a function **I**:Ω⊂ℝ^4^, where Ω is the domain of the 4D (3D space × 1D time) image and let us assume Neumann boundary conditions on δΩ, specifying the values in which the derivative of the solution is applied within the boundary of the domain. Let us now define a structure tensor **D** as a 4 × 4 symmetric and positive-definite matrix. By definition, **D** has four positive eigenvalues (λ_1_≥λ_2_≥λ_3_≥λ_4_≥0) and their associated four orthogonal eigenvectors (***θ***_1_, ***θ***_2_, ***θ***_3_ and ***θ***_4_) explain the distribution and orientation of the gradient ∇**I** = (*I*_*x*_, *I*_*y*_, *I*_*z*_, *I*_*t*_) of the image **I** in a given neighborhood. A structure tensor can distinguish between anisotropic and isotropic diffusion. If λ_1_>>λ_2_, λ_3_ and λ_4_, the structure tensor has a principal orientation (in this case ***θ***_1_) and the diffusion is anisotropic. It can be represented with an ellipsoid oriented along ***θ***_1_. On the other hand, if λ_1_≈λ_2_≈λ_3_≈λ_4_, the structure tensor is not oriented in a main direction and ***θ***_1_, ***θ***_2_, ***θ***_3_, and ***θ***_4_ are eigenvectors of **D** with equal weight. In this situation, the diffusion is isotropic and the structure tensor can be represented with a sphere.

Inspired by the physical process of diffusion, we link the diffusion to fMRI image regularization and we propose the A4D-fMRI for the enhancement of coherent structures found in fMRI data. The A4D-fMRI recovers brain activations and smooths small variations while preserving large variations *via* a regularization that is applied on the 4D fMRI image, acting simultaneously in the 3D space and the 1D time dimensions. Using this approach corresponds to performing minimization of image variations as well as a blind image deconvolution. To do this, we propose a regularization process based on a gradient descent computed with PDEs, such that


(3) $$\frac{\partial \text{I}}{\partial t}=(1-\alpha )\frac{{\text{H}}^{T}({\text{I}}_{0}-\text{HI})}{\|{\text{I}}_{0}{\|}_{2}}+\alpha \frac{\text{div}(\tilde{D}\nabla \text{I})}{\|\text{div}({\tilde{D}}_{0}\nabla {\text{I}}_{0}){\|}_{2}}$$

where the term on the left, ∂**I**/∂*t*, is the regularization flow, the first term on the right is the data fitting term, also called fidelity term, that prevents the solution from straying far from the input data, and the second term on the right minimizes image variations. The parameter α∈[0, 1] is the user-defined regularization parameter that balances the fidelity and the regularization terms. Starting from the initial image **I**_0_, the restored image **I** is regularized as *t* increases reducing noise and extracting coherent space and time variations. At the same time, no new structures are introduced in the image (Aubert and Kornprobst, 2006).

Going more into the details of the fidelity term


(4) $$F(\text{I})=\frac{{\text{H}}^{T}({\text{I}}_{0}-\text{HI})}{\|{\text{I}}_{0}{\|}_{2}}$$

where **I**_0_ and **I** are the original and the regularized image, respectively, ||**I**_0_||_2_ in the denominator is the normalization factor, **H** is the HRF (Khalidov et al., 2011) operator, and **H**^*T*^ is its transpose. The multiplication of **H**^*T*^ with (**I**_0_−**HI**) corresponds to a correlation and can be implemented *via* convolution with the time-reversed HRF. Note that this product is computed only along the time dimension. The HRF considered in this work is the linearized time-HRF operator proposed by Friston et al. (2000) and Khalidov et al. (2011).

The regularization term is defined as


(5) $$R(\text{I})=\frac{\text{div}(\tilde{D}\nabla \text{I})}{\|\text{div}({\tilde{D}}_{0}\nabla {\text{I}}_{0}){\|}_{2}}$$

where $\|\text{div}({\tilde{D}}_{0}\nabla {\text{I}}_{0}){\|}_{2}$ is the normalization term and $\tilde{D}$ is the regularization tensor, distinct from the structure tensor **D**. In order to elucidate the regularization term, let us start by the definition of the operator


(6) $$\text{D}=\frac{\nabla I\nabla I^{T}}{\|\nabla I{\|}^{2}}*G$$

that is the 4D structure tensor of **I** smoothed by the Gaussian kernel *G* with standard deviation σ_*G*_ *via* the convolution operator *. The matrix **D** being the diffusion tensor of the image **I**, its eigendecomposition gives a set of eigenvalues and eigenvectors such that, if the gradient in one direction is large, the eigenvalue associated with that direction is large, whereas the eigenvalues associated with the other three directions are relatively small. Since we are processing fMRI images with the aim of saving activations and contours that occur concomitant to a large gradient in a certain direction, we aim at reversing the diffusion process, therefore at reversing the effect of **D** into $\tilde{\text{D}}$ to enhance and simultaneously simplify coherent structures of the fMRI image. Here, we propose to compute the operator $\tilde{D}$ by modifying the eigenvalues of the operator **D** in Equation (6). Specifically, we defined the directions of the image variations by an eigendecomposition of **D** such that


(7) $$\text{D}=\text{Q}\Lambda {\text{Q}}^{T}$$

where **Q** contains the orthogonal eigenvectors (***θ***_1_, ***θ***_2_, ***θ***_3_, ***θ***_4_) of **D** and **Λ** contains their associated eigenvalues (λ_1_≥λ_2_≥λ_3_≥λ_4_). We then recomputed the matrix


(8) $$\tilde{D}=\text{Q}\tilde{\Lambda }{\text{Q}}^{T}$$

where $\tilde{\Lambda }$ is a diagonal matrix with entries ${\tilde{\lambda }}_{1}$, ${\tilde{\lambda }}_{2}$, ${\tilde{\lambda }}_{3}$, and ${\tilde{\lambda }}_{4}$ such that for each voxel the highest eigenvalue is given by


(9) $${\tilde{\lambda }}_{1}=\text{exp}(-\frac{\lambda_{1}^{2}}{\text{max}{\left(\Lambda \right)}^{2}}\frac{1}{2\sigma_{D}^{2}})$$

and the other eigenvalues are ${\tilde{\lambda }}_{2}={\tilde{\lambda }}_{3}={\tilde{\lambda }}_{4}=1$. The rationale is that, if λ_1_ is large, the current voxel may be located on a edge or activation and the diffusion tensor $\tilde{D}$ is steered to be anisotropic, by setting ${\tilde{\lambda }}_{1}\ll {\tilde{\lambda }}_{2},{\tilde{\lambda }}_{3},{\tilde{\lambda }}_{4}$. Since we aim at performing a smoothing only along the other three directions to smooth preferably along the coherence directions, the three eigenvalues λ_2_≈λ_3_≈λ_4_ are set to 1. On the other hand, if λ_1_ is small, the diffusion will be isotropic in the four directions because ${\tilde{\lambda }}_{1}\approx 1$ and ${\tilde{\lambda }}_{2}={\tilde{\lambda }}_{3}={\tilde{\lambda }}_{4}=1$. Using the function in Equation (9) corresponds to reassigning to each voxel different eigenvalues constituting the matrix $\tilde{\Lambda }$, before recomputing the operator $\tilde{D}$ as in Equation (8). In fact, if λ_1_/max(**Λ)** is large, the highest eigenvalue $\tilde{\lambda_{1}}$ of the considered voxel will tend to zero. This steers the geometrical regularization to be anisotropic, because the smoothing will apply equally in the remaining three directions but it will be negligible in the normal to the detected contour. Otherwise, if λ_1_/max(**Λ)** is small, the greatest eigenvalue $\tilde{\lambda_{1}}$ will tend to 1 and this leads to an isotropic regularization almost in all the four directions (x, y, z, t). In both cases, $\tilde{\lambda_{2}}$, $\tilde{\lambda_{3}}$, $\tilde{\lambda_{4}}$ are set to 1. This procedure is applied to each voxel of the entire 4D image such that at each iteration the image **I** computed in Equation (3) is removed from the image at the previous iteration.

In practice, the gradient and divergence operator are implemented using finite differences. The gradient was implement using a left to right scheme and the divergence using right to left, leading to a discrete Laplacian if the diffusion tensor was omitted. To maintain stability across iterations used to numerically solve the PDE, the step size must be small, but reducing it increases computation times. We empirically set it to 0.1, which provided stability and acceptable computation times.

In this way, supposing the brain activates in constant blocks, we regularized the image together in space and time. We were able to keep large image variations occurring during brain activations or spatial edges, and to gradually remove small variations, corresponding to noise, while conserving and enhancing coherent structures of the fMRI image. The theoretical framework of the A4D-fMRI explained above has been implemented in a Python package that can be downloaded at the following link: gitlab.inria.fr/cobcom/a4dfmri. The validation illustrated in the following sections is performed using the A4D-fMRI package.^1^

## 3. Methods

### 3.1. Simulation of fMRI Data

To reproduce the acquired fMRI signals, we started by simulating the fMRI activation as a piece-wise constant function as proposed by Farouj et al. (2017). For each voxel *v*, we modeled the activity-inducing signal as a boxcar function


$$\begin{array}{l} u\left(t\right)=H\left(t-a\right)-H\left(t-b\right) \end{array}$$

where *H*(*t*) is the Heaviside step function. We added noise to *u*(*t*) representing the random intrinsic electrical fluctuations within neuronal networks that are not associated with encoding a response to internal or external stimuli. To do this, we corrupted the activity-inducing signal *u*(*t*) with an additive random Gaussian noise with zero mean and standard deviation σ_*m*_ that we called “model noise” ϵ_*m*_. The noisy activity-inducing signal is


$$\begin{array}{l} u_{n}\left(v,t\right)=u\left(t\right)+\epsilon_{m}. \end{array}$$

We modeled the activity-related signal *x*(*t*), consequent to the neural activation as the convolution of *u*_*n*_(*t*) with the HRF, modeled as the linear time-invariant system *h*(*t*) (Khalidov et al., 2011):


$$\begin{array}{l} x\left(v,t\right)=u_{n}\left(v,t\right)*h\left(t\right). \end{array}$$

**Figure 1:**
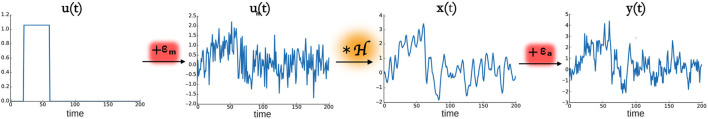
fMRI model. From left to right, *u*(*t*) is the activity inducing signal that represents the neural activation as a piece-wise constant signal. To this signal, a model noise ϵ_*m*_ with a Gaussian distribution was added, thus leading to the signal *u*_*n*_(*t*) that was then convolved with the hemodynamic response function operator H, thus obtaining *x*(*t*), also called activity-related signal. Adding the noise ϵ_*a*_ to the signal *x*(*t*) gives the simulated acquired fMRI data denoted as *y*(*t*).

Real time series acquired using the fMRI technique is corrupted by different kinds of noise and artifacts given by mechanisms that do not reflect any neurophysiological function, such as heart rate, respiratory fluctuations, motion artifacts, thermal noise, and scanner drifts (Lund et al., 2006). For this reason, we added noise to *x*(*t*) thus obtaining the acquired fMRI signals


$$\begin{array}{l} y\left(v,t\right)=x\left(v,t\right)+\epsilon_{a}=u_{n}\left(v,t\right)*h\left(t\right)+\epsilon_{a} \end{array}$$

where ϵ_*a*_ is the additive random Gaussian noise with zero mean and standard deviation σ_*a*_. A scheme representing the model of the phantom fMRI data is shown in Figure 1.

### 3.2. Validation of Synthetic Data

**Figure 2:**
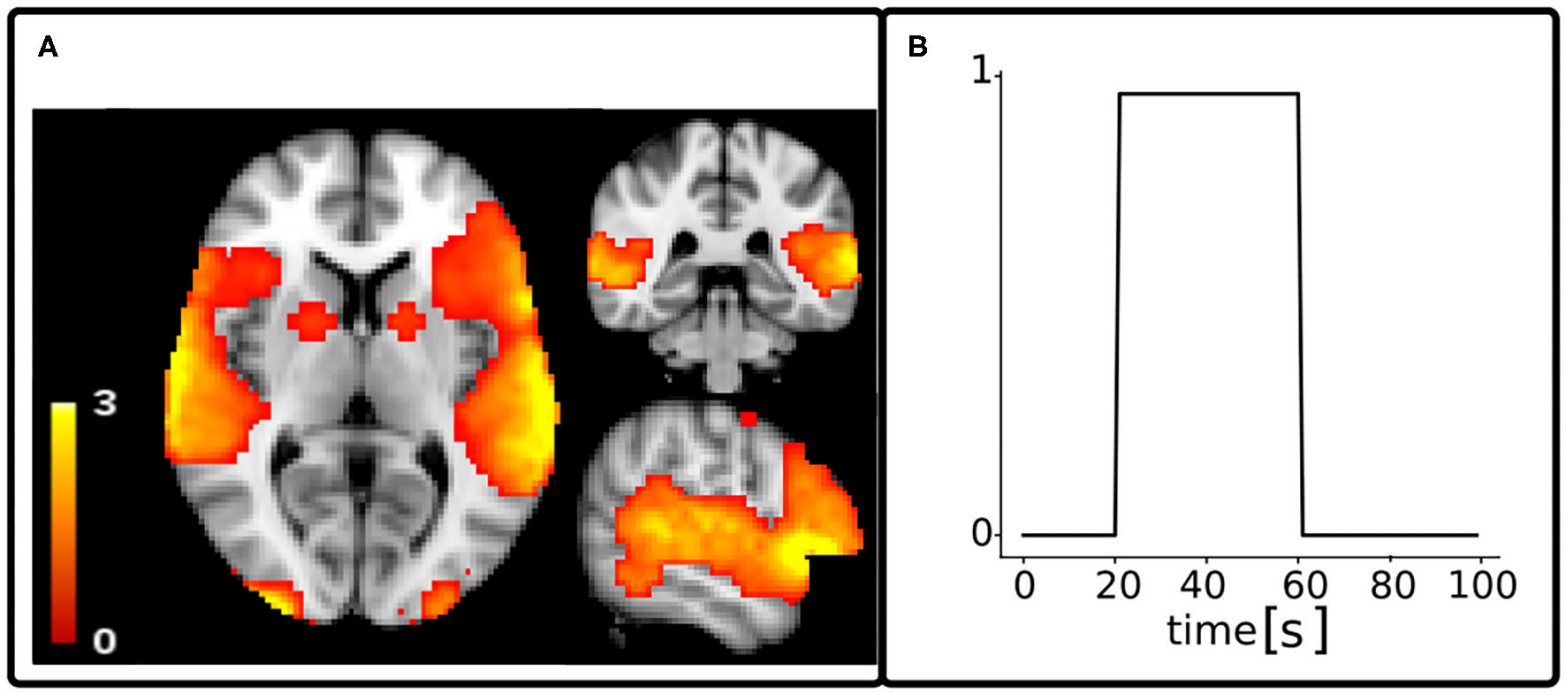
Spatial map **(A)** and time series **(B)** of the activation considered as ground truth for functional MRI simulated data. The time course of the activation *u*(*t*) was simulated with a repetition time of 1 s.

To test and validate the A4D-fMRI, we scaled a 3D activation map computed with the FMRIB Software Library (FSL^2^) Physics-Oriented Simulated Scanner for Understanding MRI (POSSUM^3^) in the range [0, 3], with a 2-mm isotropic resolution (Figure 2A). We multiplied it by a piece-wise constant signal *u*(*t*) of 100 s, with one onset of 40 s, from 20 to 60 s (Figure 2B). Starting from *u*(*t*), we simulated the acquired fMRI time-courses *y*(*t*) as explained in the previous section. We tested the A4D-fMRI on several simulated images obtained by adding different amount of noise for each experiment. We regularized the whole image using the A4D-fMRI as shown in Section 2, and we recovered the voxel-wise activity-inducing signals û(*t*). To evaluate the results, we compared the simulated activity-inducing signal *u*(*t*) with the recovered û(*t*). To do this, for each voxel's time course belonging to the GM, we first computed the mean square errors (MSEs) between û(*t*) and *u*(*t*). Second, we computed the roots of the mean (RMSE) and the standard deviation (STD) of this array set. To be able to compare these values to the TA results, which restrict computations to the Gray Matter (GM) voxels, we only considered values obtained among the voxels belonging to the GM mask. Similarly, the Pearson correlation (*r*) was computed for each GM voxel between the simulated activity-inducing signal *u*(*t*) and the recovered û(*t*). The mean and the STD was than computed on this set of *r* values. We compared our results with those obtained using the TA approach (Farouj et al., 2017), implemented in the TA toolbox.^4^ Note that because of the L1-norm regularization used by TA which tends to underestimate signal amplitudes, comparing the results based only on amplitudes is not fair (see Supplementary Materials for additional discussion). For this reason also, we compared the performance of both algorithms based on the correlation between the simulated and the recovered activation. Pearson's correlation coefficient is invariant to the scale of the input signals and therefore ignores the signal shrinkage associated with the L1-norm and quantifies only the shape of reconstruction. We did not explicitly set any regularization parameters for the TA approach and instead relied on the automatic selection heuristics provided by the implementation of the TA toolbox^4^ (Karahanoğlu et al., 2013; Farouj et al., 2017). The results presented were obtained by providing only our data with its specific repetition time (TR). The temporal regularization parameter is initialized in the TA toolbox based on a pre-estimated noise level value of the data fit, derived from the median absolute deviation of fine-scale wavelet coefficients, then updated at each iteration. The spatial temporal regularization parameter is already preset in the TA toolbox for the gray matter constrained total variation algorithm and therefore follows the recommendation of the authors. To investigate the performance of our algorithm to event-related designs, we also simulated brain activations as spikes and applied our approach on the noisy synthetic fMRI images.

### 3.3. Validation of Real Experimental Data

#### 3.3.1. Task-fMRI Data

In addition to validating the A4D-fMRI on simulated data, we evaluated its performance on real data. Although our approach was conceived to be applied to rs-fMRI data, or in any situation where no experimental paradigm is given, we test it here on task-fMRI data. This testing strategy provides a ground truth, i.e., the task timing, which can be used to assess the performance of the algorithm.

The study was conducted on the motor task-fMRI data from 51 subjects from the Human Connectome Project (HCP) database (Van Essen et al., 2013). The data underwent a minimal pre-processing pipeline (Glasser et al., 2013), which includes correction of gradient-non linearity-induced distortions, registration of each image frame to the signal-band reference image to achieve motion correction, phase-encoding distortion correction, EPI image distortion correction, registration of the fMRI volumes to the structural data, coregistration of the fMRI data to the Montreal Neurological Institute (MNI) space, masking and fMRI image intensity normalization to the 4D whole global mean of 10^3^. As additional pre-processing steps, each voxels' time course was detrended to remove linear trends and normalized to 0 mean and unit standard deviation. The motor task is initiated by a visual cue followed by the movement of the left and right foot, the left and right hand, and the tongue. The tasks starting points were considered equal for each subject and inter-subjects differences of the order of milliseconds were neglected.

After applying the A4D-fMRI on the entire brain images of each subject, we recovered the reconstructed activity-inducing signals û(*t*) without prior knowledge on the onset/offset times and location of the evoked stimuli. The regularization parameter α was set experimentally to 0.9997, σ_*G*_ was set to 1, σ_*D*_ was set to 0.2, and we computed up to 40 iterations. The value of α is driven by two factors: the divergence of the expected solution and the amount of noise in the data. One is very small converging toward zero and a second one stabilizing at the variance of the noise in the data. This stopping criteria was chosen because it was the minimal number of iterations required for the Pearson correlation to stabilize for all tasks fMRI datasets.

To highlight the ability of the A4D-fMRI to recover brain activations without knowledge of the experimental paradigm, we qualitatively compared brain regions recovered using the A4D-fMRI to those recovered using the GLM as implemented in the FSL library. The GLM model requires as input the exact occurrence of the tasks that the subject is asked to perform. We run the A4D-fMRI on the whole brain thus blindly recovering brain activations, without prior knowledge on the intervals of the evoked stimuli. To estimate the results obtained using the A4D-fMRI, we computed the voxel-wise correlation maps, by estimating the Pearson correlation coefficient (*r*) between the recovered activations and the five tasks. The tasks were simulated as a piece-wise constant signal with unit amplitude when the subject is performing the task and zeros elsewhere. As for the GLM, we included the five tasks in a design matrix and we estimated the regressors' weights with FSL. Results showing differences and similarities of both approaches were qualitatively assessed.

Subsequently, we quantitatively compared the results obtained using the A4D-fMRI with the ones given by the TA, on the sample data composed by 51 subjects. We first defined four ROIs located in brain regions that are involved in the five considered tasks. The ROI related to the tongue was bilateral, whereas for the hands and the feet we defined separate ROIs for the left and the right side of the brain. To do this, we started by defining the ROIs from the work proposed by Roux et al. (2018), who mapped the somatosensory homunculus MNI coordinates using the electrostimulation. For each coordinate center, we built a spherical 3-mm-radius ROI and we grouped the multiple ROIs related to each task into a unique ROI. Coordinates' centers are shown in Table 1.

**Table 1:** Montreal Neurological Institute (MNI) coordinates centers of the brain areas found to respond to the somatosensory stimulation.

**Task**	**ROI location**	**MNI center coordinates [mm]**
Right\left hand	Thumb	±46.6; −22.8; 56.2
	Index finger	±43.3; −26.8; 59.9
	Middle finger	±40.8; −28.6; 62
	Ring finger	±37.5; −29.7; 64.8
	Little finger	±35.2; −30.9; 66.3
Tongue	Base	±61.4; −11.1; 23.3
	Middle	±60.7; −11.4; 30
	Tip	±59.2; −11; 36
Right\left foot	–	±4; −41; 64

*The coordinates, adapted by Roux et al. (2018), are expressed in MNI standard space*.

After defining the ROIs, similarly to the comparison between the A4D-fMRI and the GLM, we computed the whole-brain voxel-wise correlation maps between the time course related to each task and the recovered activity-inducing signals û(*t*) obtained with the A4D-fMRI and the TA. For each subject, we first computed the average of the Pearson correlation coefficients (*r*) inside the GM-masked ROIs and then calculated the mean and the standard deviation of these averaged correlation values across the 51 subjects belonging to the sample data.

Furthermore, to show that the A4D-fMRI is able to differentiate between a region that is active and one that is not, the time courses û(*t*) of one representative subject (100307) were averaged in two ROIs of 6 × 6 × 6*mm*^3^: one that is expected to be active during the task, and one located in a brain area which is not involved in the task. We selected the task related to the tongue, and we chose one ROI centered in the Brodmann Area 4p (rBA4p; MNI coordinates: 62, −14, 30) that is activated during a tongue motor task, and another centered in the primary auditory cortex (TE1.2; MNI coordinates: 56, 4, 10; Kiviniemi et al., 2003), that is not involved in the tongue movement. The Pearson correlation (*r*) was computed between the tongue activation and the recovered û(*t*) for each voxel, and then averaged among the voxels belonging to the two GM-masked ROIs. Again the tongue activation was simulated as a piece-wise constant signal with unit amplitude during the task and zeros elsewhere. We compared results obtained using the A4D-fMRI with the ones obtained using the TA toolbox.

#### 3.3.2. Resting-State fMRI Data

Finally, we applied the A4D-fMRI on the rs-fMRI image of one subject (100307) from the HCP database. The data were acquired with a SIEMENS MAGNETOM Connectome Syngo MR D11 using a gradient-echo EPI sequence (TR = 720 ms; TE = 33.1 ms; flip angle = 52°; FOV = 208 × 180 mm; slice thickness 2.0 mm; number of slices = 72; 2.0 mm isotropic voxels; multiband factor = 8). The subject was asked to lay in the scanner without thinking about anything in particular. The number of acquired frames was 1,200 and the duration of the acquisition was 14:33 min. In the case of rs-fMRI data, the task paradigm is unavailable since the subject does not perform any task in the scanner. The data underwent the same minimal preprocessing of the task fMRI data as proposed in the HCP pipeline (Glasser et al., 2013). In addition, the time series were detrended to remove linear drifts and normalized to zero mean and unit standard deviation. We applied the A4D-fMRI algorithm on the entire rs-fMRI sample and we observed the dynamics of the recovered activation maps across time.

## 4. Results

### 4.1. Validation on Synthetic Data

**Figure 3:**
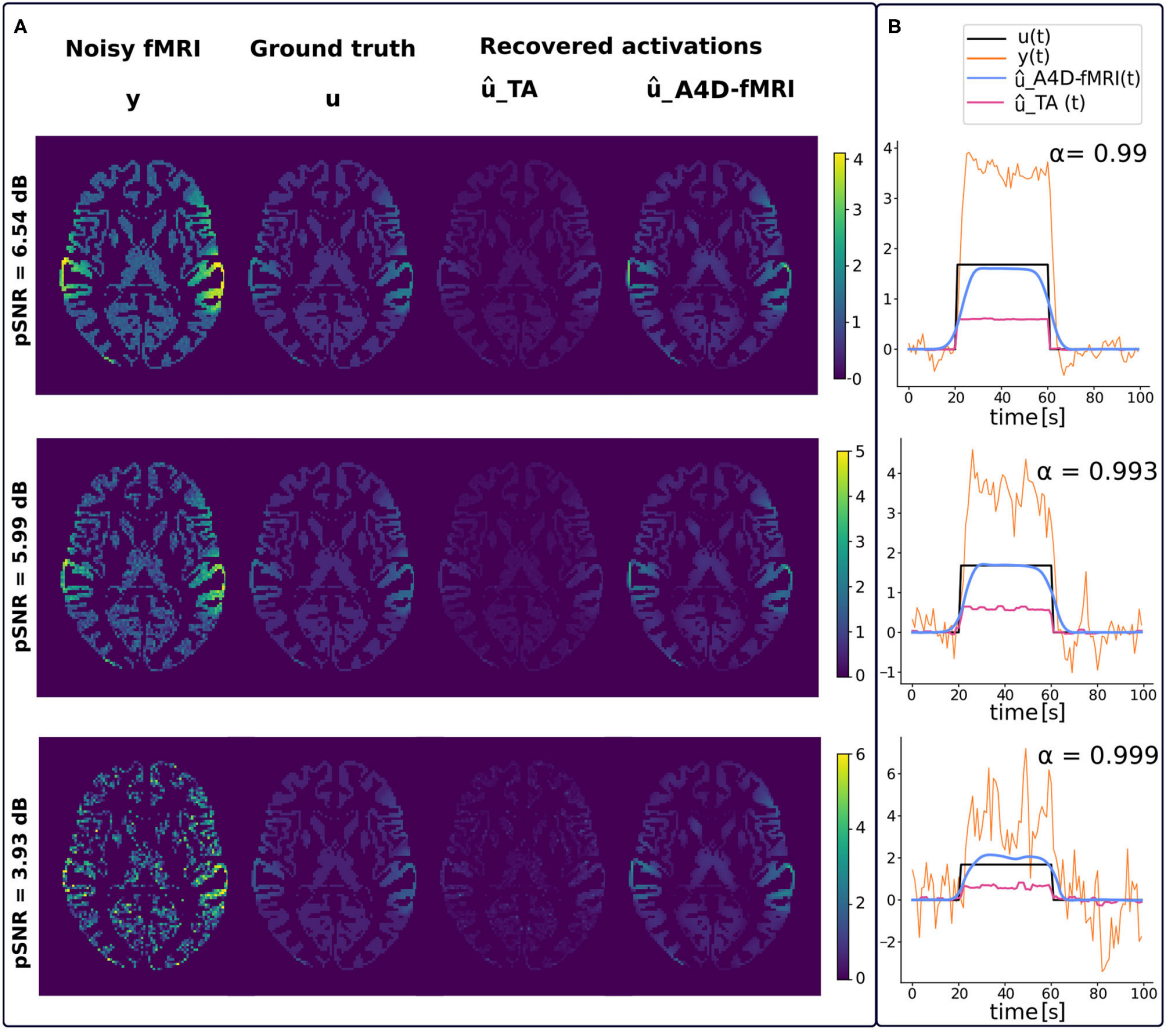
**(A)** From left to right, spatial maps of the simulated fMRI image *y*, ground truth activation *u*, recovered activation using the total activation (TA) approach (û_*TA*_) and our approach (û_*A*4*D*−*fMRI*_). Each row corresponds to a different peak-SNR (pSNR): 6.54, 5.99, and 3.93 dB from the top to the bottom. **(B)** Reconstructed time series û(*t*) obtained with our approach [û_*A*4*D*−*fMRI*_(*t*), light-blue] and the TA approach [û_*TA*_(*t*), magenta] superimposed on the activation [*u*(*t*), black] and functional MRI (fMRI) signal [*y*(*t*), orange].

Figure 3 shows examples of regularized spatial maps (Figure 3A) and time series (Figure 3B) using the A4D-fMRI (û_*A*4*D*−*fMRI*_) and the TA (û_*TA*_). Both approaches do not require any prior knowledge of the paradigm timing. The regularized spatial maps in Figure 3A represented in the axial plane show how the regularized fMRI image recovered with the A4D-fMRI (û_*A*4*D*−*fMRI*_) is closer to the ground truth (*u*) in terms of signal amplitude with respect to those obtained using the TA û_*TA*_. This is verified for different peak-SNRs (pSNRs), i.e., 6.54, 5.99, and 3.93 dB. In Figure 3B, we show examples of regularized time courses and that we recover an amplitude closer to the ground truth when compared to the method implemented in the TA tool. We also show smoother recovered signals when compared to the TA. Additional tests and results are shown in Supplementary Figure 1.

**Figure 4:**
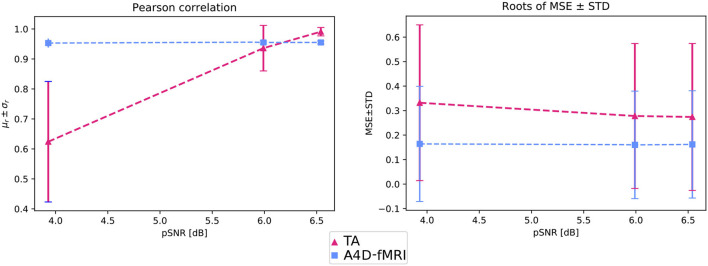
On the left, the graph shows, for different pSNRs, the mean Pearson correlation coefficients (μ_*r*_) and related standard deviation (σ_*r*_) computed between *u*(*t*) and û(*t*) and averaged among the voxels belonging to the GM. On the right, The graph shows, for different pSNRs, the roots of the mean squared errors (MSEs) and standard deviations (STDs) between *u*(*t*) and û(*t*) averaged among the voxels belonging to the GM.

Figure 4 shows that the roots of MSEs ± STDs computed between the simulated activation *u*(*t*) and the recovered one û(*t*) change for different pSNRs. We show lower errors with lower standard deviations than the ones obtained using TA.

**Figure 5:**
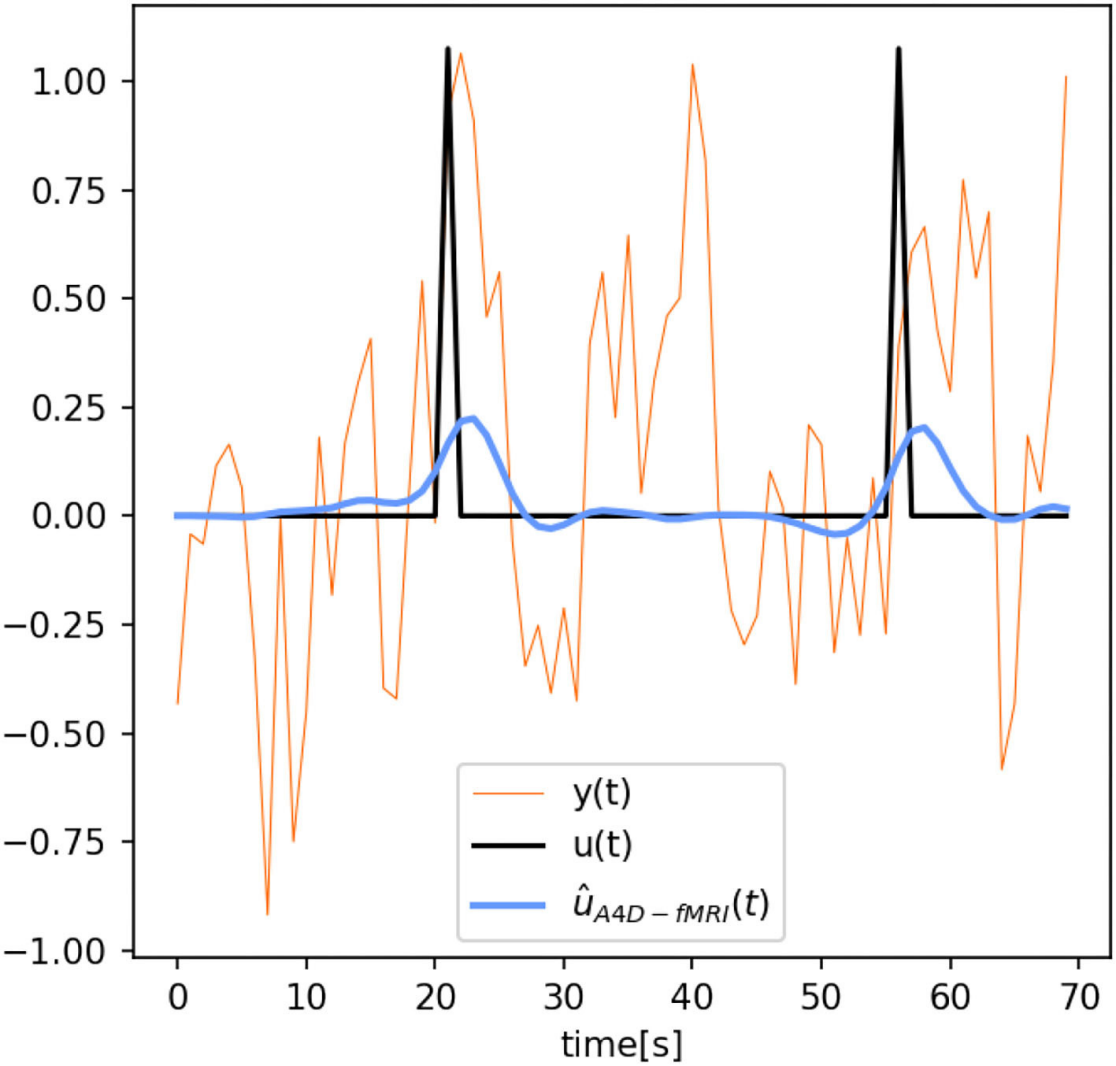
Reconstructed time series û(*t*) obtained with the anisotropic 4D-fMRI (A4D-fMRI) [û_*A*4_*D*__*f*_*MRI*_(*t*), light-blue] superimposed on the spike activation [*u*(*t*), black] and functional MRI (fMRI) signal [*y*(*t*), orange]. Peak-SNR = 13.23 dB.

Figure 4 also shows that the activation recovered with the A4D-fMRI is more correlated with the ground truth (*r*≈1) for different pSNRs. Although the results obtained with TA are more sensitive to noise and show better performances for less noisy data: the mean correlation increases according to the pSNR while the standard deviation decreases. Results related to event-related designs, where activations were simulated as spikes, are illustrated in Figure 5. The results show that we are able to recognize a spike activation and remove the noise (see Figure 5 between 30 and 50 time points). However, the obtained activity-inducing signals are smoothed and do not match the exact lengths of the simulated spikes. We refer the reader to Supplementary Figures 2, 3 to see the results for different spike trains and model and additive amount of noise.

### 4.2. Validation on Real Data

#### 4.2.1. Task-fMRI Data

**Figure 6:**
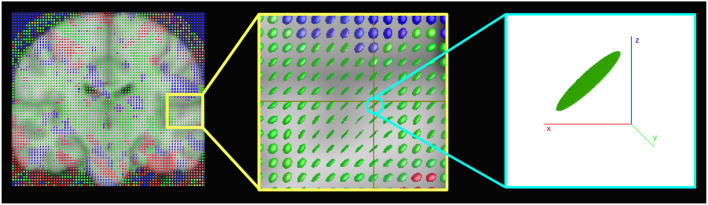
Representation of the structure tensors computed with the anisotropic 4D-fMRI (A4D-fMRI). The images show for a 3D spatial map, superimposed to the standard MNI template, how the structure tensors look like ellipsoids or spheres meaning that the geometric regularization is applied anisotropically. Images were made using MRview (Tournier et al., 2012).

Figure 6 shows the structure tensors $\tilde{D}$ (Equation 8) in a coronal brain slice for a representative subject of the HCP database. Note that the image refers to the spatial maps, and the temporal dimension is not represented. The presence of ellipsoids oriented in different directions rather than spheres shows the anisotropic nature of the regularization.

**Figure 7:**
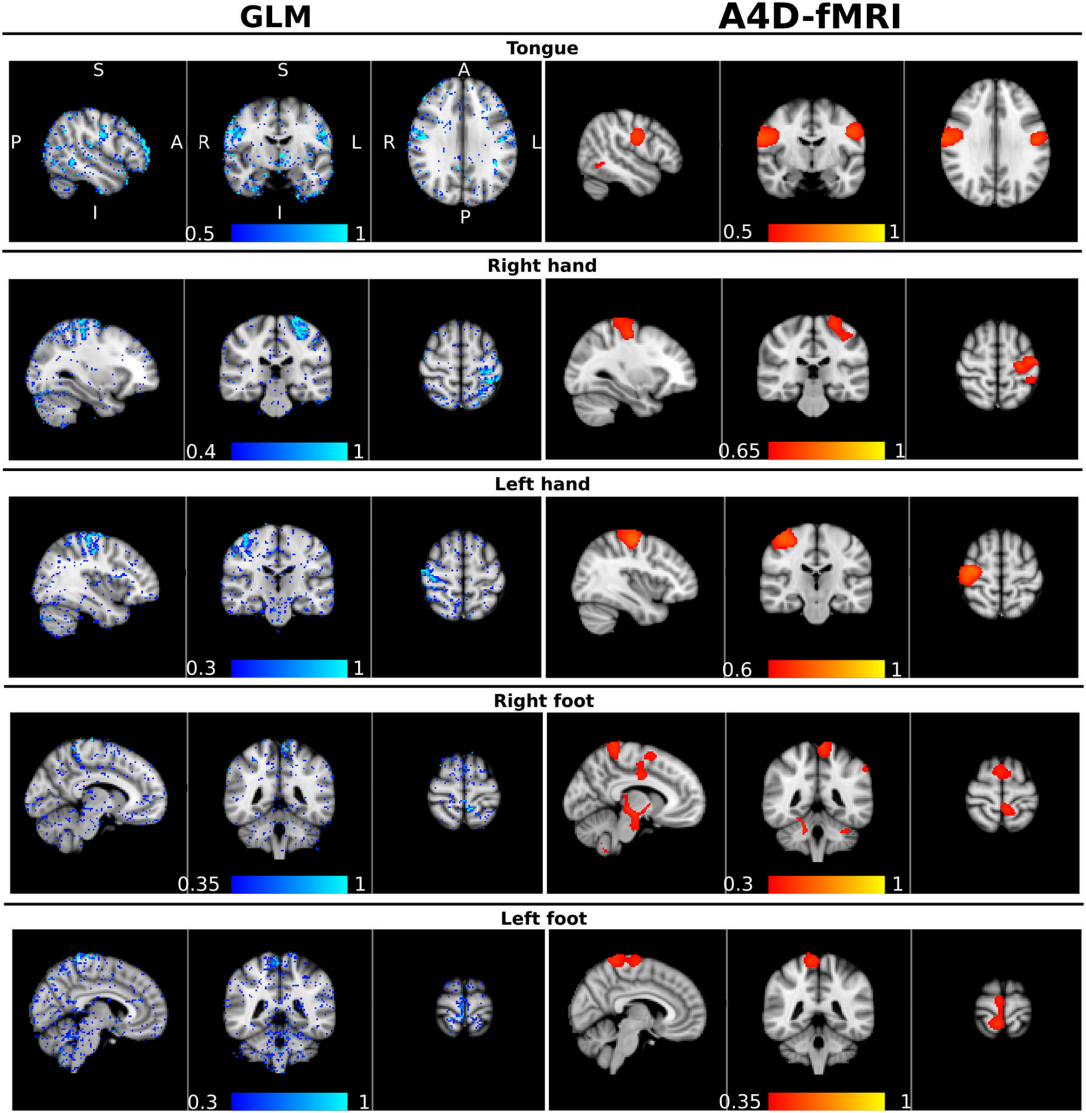
Qualitative comparison between the general linear model (GLM) and the anisotropic 4D-fMRI (A4D-fMRI). On the left column, in a blue-lightblue color-map, superimposed to the standard MNI template, the β-regressors map obtained using the GLM implemented in FMRIB Software Library (FSL). On the right column, in a red-yellow color map, the whole-brain voxel-wise correlation maps obtained using the A4D-fMRI superimposed to the standard MNI brain. The Pearson correlation (*r*) was computed voxel-wise across the whole brain, between the reconstructed activity inducing signals û(*t*) and the five motor tasks simulated as piece-wise constant signals with ones in the time points where the subject is executing the task and zeros elsewhere. The values *r* of the correlations are indicated by the color bars. Each row corresponds to a specific motor task, from the top to the bottom: the tongue, the right and left hand, and the right and left foot. A, anterior; P, posterior; S, superior; I, inferior; R, right; L, left. The unthresholded images are available in Supplementary Materials.

As for the real data analyses, and specifically the comparison between the A4D-fMRI and the GLM, we show that the correlation maps related to each task computed with the A4D-fMRI were well overlapped to the values of the regressors coefficients obtained using the GLM as shown for one illustrative subject (100307) in Figure 7. The GLM shows results that follow the GM, while the activations found with the A4D-fMRI, which again were performed across the whole brain, and not masked with the GM mask, cover also voxels across the white matter. Interestingly, the found activations overlap the areas found to be active in the motor homunculus brain (Penfield and Boldrey, 1937).

**Figure 8:**
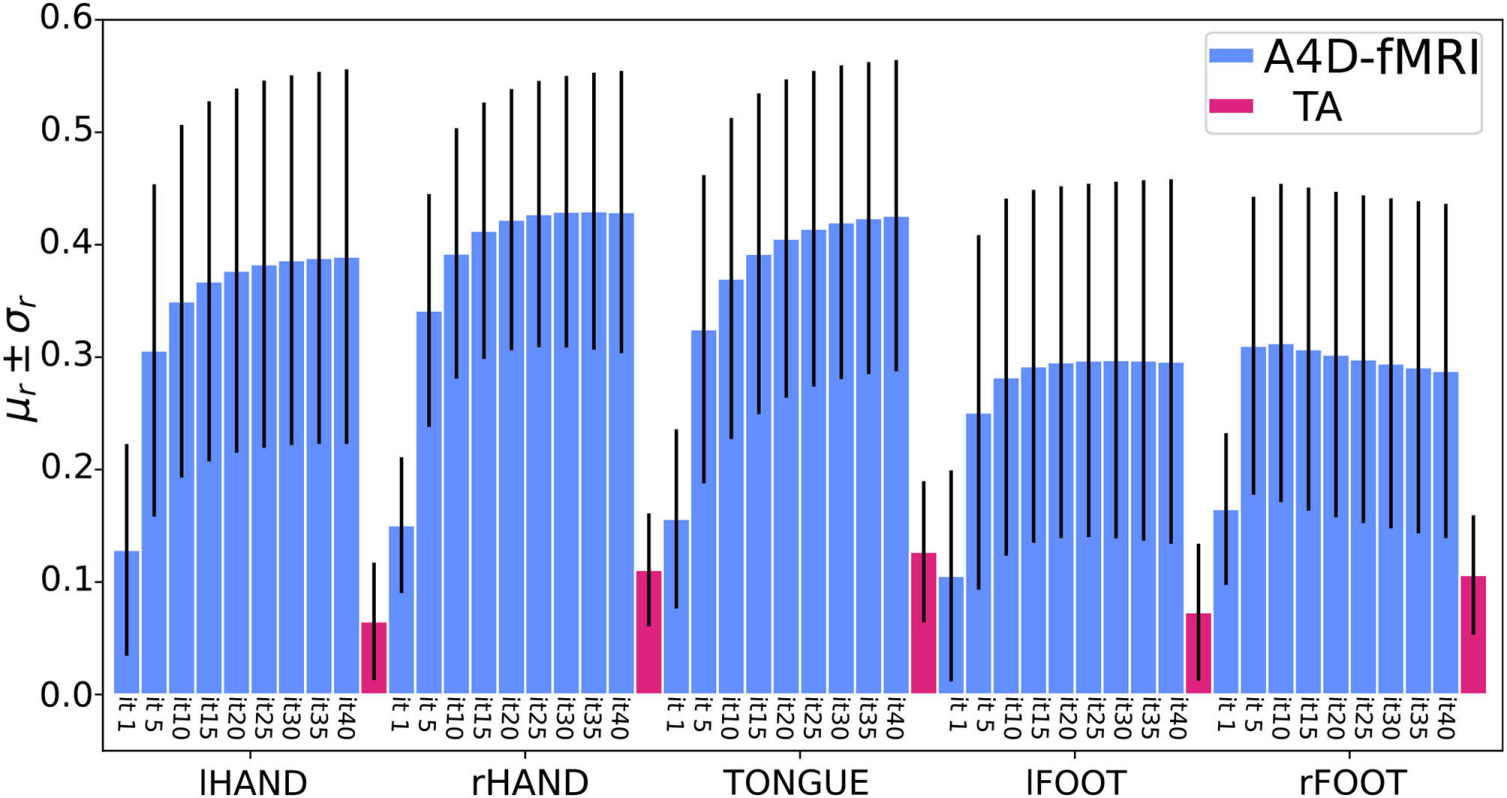
Barplots of the mean (μ_*r*_) ± standard deviations (σ_*r*_) of the Pearson correlation coefficients (*r*) computed on the sample data (51 subjects) in five regions of interests (ROIs) related to the tasks of the left and right hand, the tongue, and the left and right foot. For each task, the bars in light-blue represent the results using the anisotropic 4D filtering fMRI (A4D-fMRI) for an increasing number of iterations (from 1 to 40). The bars in magenta represent the results using the total activation (TA) toolbox. (lHAND, left hand; rHAND, right hand; lFOOT, left foot; rFOOT, right foot).

**Figure 9:**
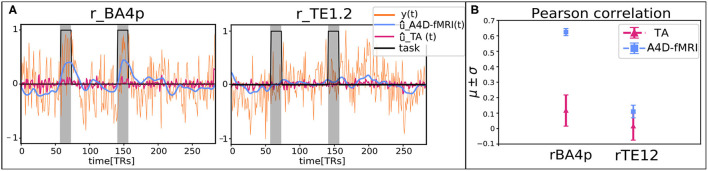
**(A)** Reconstructed signals û(*t*) obtained with the anisotropic 4D filtering fMRI (A4D-fMRI) (light-blue) and the total activation (TA) tool (magenta) superimposed on the real acquired functional MRI (fMRI) signals (orange) and the simulated tongue activation (black). The plot on the left is related to the regions of interest (ROI) located on the Brodmann Area 4p (rBA4p), the plot on the right is associated to the ROI positioned on the primary auditory cortex (rTE1.2). All the signals were averaged across the voxels belonging to the GM-masked ROIs. The gray areas represent the occurrence and the duration of the tongue movements. **(B)** Mean Pearson correlation coefficients (μ) and their associated standard deviations (σ) computed between the tongue activation and the recovered signals û(*t*) averaged across the voxels belonging to the GM-masked ROIs (rBA4p on the left, rTE1.2 on the right).

Quantitative comparison between the activity-inducing signal recovered using the A4D-fMRI and the TA is shown in Figure 8. Results show that the mean Pearson correlation values estimated for each ROI across the data sample increase while increasing the number of iterations, until it converges after 25 iterations for the hands, 5 iterations for the feet, and about 35 iterations for the tongue. Moreover, starting from the first iteration, we show statistically significant higher correlation values compared to the ones obtained using the TA. In particular for the comparison between the A4D-fMRI and the TA, Figure 9 shows the reconstructed signals û(*t*) (Figure 9A) and the correlations values (Figure 9B) for a single subject (100307). We show a clear difference between the correlation values estimated in the area involved in the task and the one that is not involved. In fact, we show higher correlation between the tongue activation and the recovered activation û(*t*) in the ROI rBA4p, which was expected to be involved in the motor task, while a low correlation is shown in the ROI rTE12 that instead is not involved. The TA was not able to clearly distinguish between an active and an inactive region since it showed low correlation values for both ROIs. Figure 9 clearly show another advantage of using the A4D-fMRI over TA in the fact that the recovered amplitude is higher for A4D-fMRI than TA. If a single threshold is applied on the output of the A4D-fMRI to define either the region is active or not, we are clearly able to make this distinction using the A4D-fMRI. This is not the case if a threshold is applied on the TA output, because for a low threshold both regions would be considered active, whereas for a high threshold both would result inactive. We compared the performance of the implementations of TA and A4D-fMRI by running both algorithms on the same hardware. We used the fMRI motor task data analysis with a size of 109 × 91 × 109 × 284 where 284 is the number of volumes and 109 × 91 × 109 is the MNI space dimension. TA, as implemented in the TA toolbox, and constrained in the GM voxels, ran in for approximately 9–9.5 h while our algorithm processed the whole dataset in 7 min per iteration, with a total computation time of 4.5–5 h for 40 iterations.

#### 4.2.2. Resting-State fMRI Data

**Figure 10:**
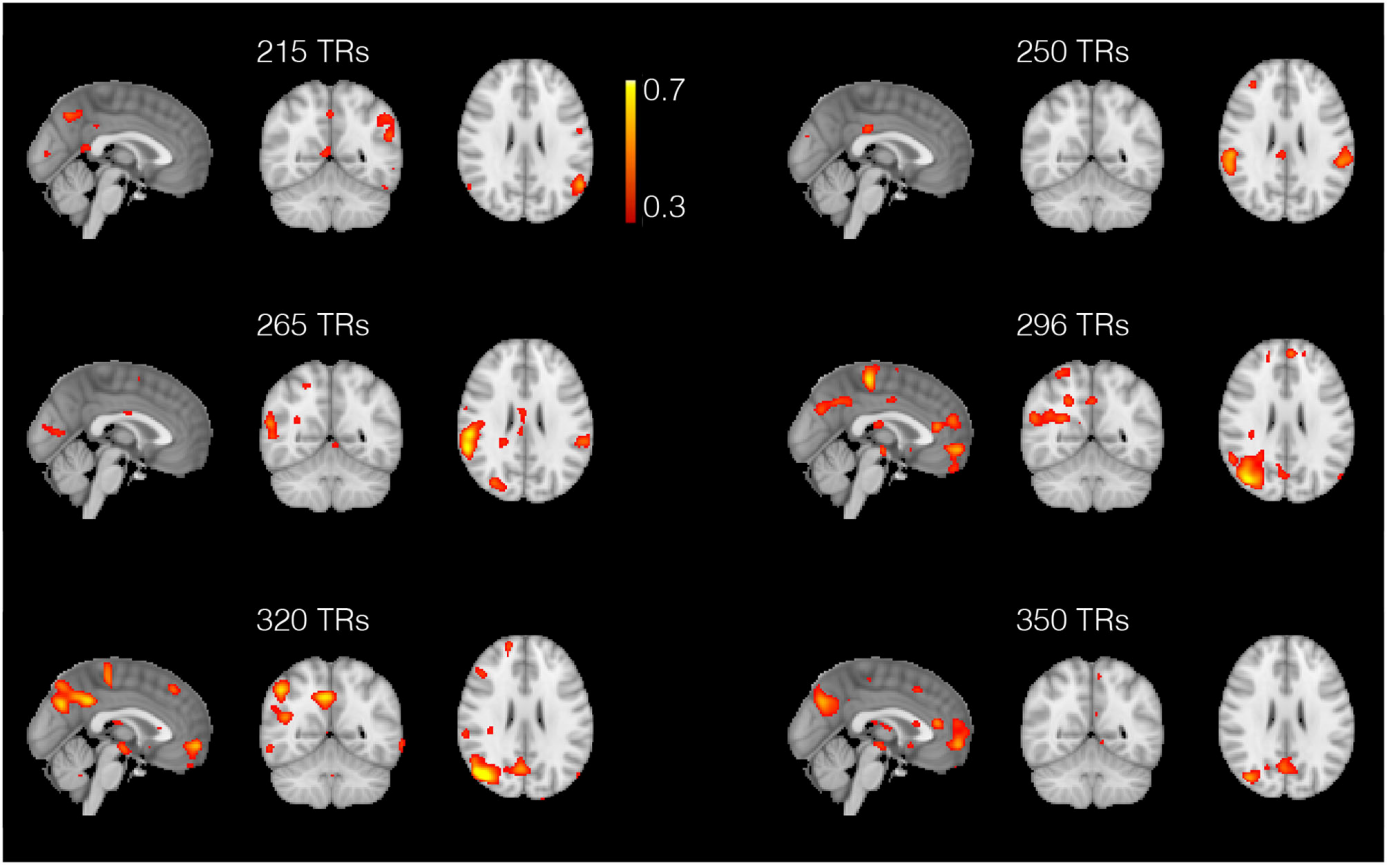
Spatial maps obtained with the anisotropic 4D filtering fMRI (A4D-fMRI) spanned across 150 repetition times (TRs) (from 200 to 350 TRs). The maps show the dynamics between the areas composing the default mode network. These regions are not all active at the same time but dynamically assemble and disassemble over time. The maps show how the lateral parietal lobules are not always active simultaneously as well as the medial prefrontal cortex that can activate selectively with the posterior cingulate cortex and the right temporal lobe.

As for the resting-state data analysis, in Figure 10 we show few example spatial maps extracted from the 1,200 analyzed time points. The figure shows a dynamic between the regions composing the default mode network, i.e., the posterior cingulate cortex, the medial prefrontal cortex, the lateral parietal lobules, and the temporal cortex (Buckner et al., 2008). As shown in the figure, these areas are not all active simultaneously, but they assemble and disassemble over time in different combinations.

## 5. Discussion

In this paper, we have described an innovative method to analyze fMRI images and recover the location and the occurrence in time of the functional neural activations. The proposed approach achieves this blindly, without the necessity of a priori knowledge of timing, duration, and location of the underlying activations. The approach we proposed, namely the A4D-fMRI, geometrically regularizes the fMRI image such that it saves and highlights large image variations as they are present at the occurrence of a brain activation or in the presence of a spatial edge with respect to small image variations that instead are removed to reduce noise. To do this, we used the PDEs in a iterative algorithm and exploited the 4-D image structure tensor that defines the directions of the gradient in the neighborhood of a voxel and directs toward an anisotropic or isotropic regularization. This gradient contains all the four principal directions of the fMRI image that is composed of a 3D spatial image repeatedly acquired in time, which suggests that the whole 4D fMRI image was smoothed contemporaneously in space and time at once.

Other approaches have been proposed to analyze fMRI data. Among them are the (i) the GLM that fits a linear model to the fMRI time series, but it assumes prior knowledge of the tasks (Friston et al., 1998); (ii) deconvolution methods, which are used to uncover brain activations from the BOLD response without prior information on the underlying activity (Gaudes et al., 2011; Caballero Gaudes et al., 2013; Karahanoğlu et al., 2013; Farouj et al., 2017). The deconvolution approach in Karahanoğlu et al. (2013) splits the problem into a spatial and temporal regularization problems, meaning that the user has to specify two regularization parameters and the two weights used to have a solution that is given by a weighted sum of the two separate regularization processes. In contrast, the A4D-fMRI overcomes several limitations found in the previous literature. When comparing the regions recovered using the GLM and A4D-fMRI, we noted overall very good agreement between the two methods. Moreover, the activations found using our approach are bigger than those found using the GLM. Both approaches found active voxels outside the GM. The A4D-fMRI was run on the entire brain volume and did not rely on a GM mask. To reduce the size of the recovered action, a GM mask can be applied to the recovered brain activation. However, in this paper, whose aim was presenting the novel algorithm, we omitted this step to show the potentials of our approach, capable of running on a whole 91 × 109 × 91 × 1,200 sized image taking only few minutes per iteration. Future developments could employ the application of a GM-mask for a functional activation analysis, or not if the interest is to investigate white matter functional activation. It should again be emphasized that, while the GLM requires knowledge of the experimental paradigm, the A4D-fMRI does not. These results highlight that the A4D-fMRI can be used to recover brain activity in the absence of an experimental paradigm, such as rs-fMRI. When comparing correlation maps obtained for the A4D-fMRI and the TA, correlation values obtained with the A4D-fMRI were significantly higher than those obtained with the TA suggesting an improved recovery of brain activity.

Compared to previous approaches, A4D-fMRI considers both the spatial and temporal nature of the fMRI signals simultaneously. This not only leads to an elegant formulation, but also allows the algorithm to leverage the notion smoothness across dimensions. For example, a brief activation of a volume of the brain or a long activation of a small area would both be detected as they correspond to a 3D subset of the 4D data. The algorithm also allows efficient implementation, which leads to reduced computation times when compared to existing strategies. Finally, while we only considered the notion of connectivity the natural representation of fMRI leading to a 4D algorithm, long-range connectivity could potentially be added by increasing the number of dimensions. This could, for example, lead to non-local filtering tied to white matter connectivity obtained from diffusion MRI and embed the notion of brain network naturally.

The A4D-fMRI can be used for different purposes, for example to recover brain activations in a task experimental paradigm as well as in a rs-fMRI study, where the subject is asked not to perform any task while lying in the MRI scanner. Hence, the A4D-fMRI could be useful to analyze the functional brain activity for those subject affected by neurological diseases that make them unable to perform a task or to analyze unexpected brain activities, for example in the case of epilepsy, thus improving the recovery of brain dynamics for future clinical applications. The A4D-fMRI could help in the recovery of time series and spatial maps that could be post-processed afterwards to perform statistical analyses. Another application of A4D-fMRI is the detection of innovation-driven co-activation patterns (iCAPs) (Karahanoğlu and Van De Ville, 2015). Indeed, iCAPs identifies spatially overlapping activation maps that reveal transients in spontaneous neural temporal activity on rs-fMRI data. It has been demonstrated that decomposing rs-fMRI data using iCAPs reveals the spatiotemporal dynamics below the so-called resting-state networks. Within this context, A4D-fMRI could be used to recover the innovation signal, which is derived from the recovered brain activations. A second application of interest is structure–function mapping (Deslauriers-Gauthier et al., 2020b) where A4D-fMRI would serve as a preprocessing step. This could potentially enhance the signal to noise ratio of the functional connectivity matrices and improve the training of models used to predict function from structure. It has been shown that including fMRI information from the A4D-fMRI approach to find active regions to be exploited as priors on cortical regions allows to select plausible structural connections to yield a tractable optimization problem to infer white matter information flow (Deslauriers-Gauthier et al., 2020a). Results, and specifically the ones shown in Figure 10, show the great potentials of the A4D-fMRI to analyze the dynamics of rs-fMRI data (Preti et al., 2017) on a larger sample in healthy control as well as in a patient group. The A4D-fMRI could also be exploited in a framework that employs the dynamic causal modeling (Friston et al., 2003). It must be clarified that the A4D-fMRI does not assume any hypothesis on the interactions between brain regions. Once the activations are inferred using the A4D-fMRI, the dynamic causal modeling could be exploited on the recovered signals to reveal possible causality between brain regions' activity. Hence, the information flow can be inferred between cortical regions known to be active using the A4D-fMRI that allows to blindly identify active regions, without requiring a manual selection of the regions of interest. A drawback of A4D-fMRI is its tendency to smooth the boundary of activity inducing signal as seen in Figure 3, particularly when compared to l1-norm approaches such as TA. It is caused by the local smoothing of the 4D tensor embodied in the parameter σ_*G*_. However, this is a necessary step of the algorithm as it improves the local estimation of the tensor and ensures it is full rank. A second source of smoothing is caused by our choice to set three eigenvalues of the modified structure tensor to 1. Filtering can therefore only be prevented in one direction, which is ideal for many signal boundaries. However, this introduces smoothing in certain cases where ideally fewer eigenvalues should be set to 1 (one such example is the spike train presented in Section 3). We are currently investigating strategies that would allow anisotropic filtering in an adapted number of directions and reduce over-smoothing.

Future works could include into the solution of the inverse problem the information given by the diffusion MRI data that would provide us with a more complex neighborhood defined by white matter connectivity. In this way, the neighborhood would no longer be given only by the surrounding voxels, but also by the voxels which are anatomically segregated and therefore functionally connected to achieve the same function. In addition, the proposed A4D-fMRI approach could be exploited to investigate possible fMRI activations in the white matter, which is an emerging debated topic in the neuroimaging field (Huang et al., 2018).

## 6. Conclusion

In this article, we proposed and validated a new method to blindly regularize the fMRI images and recover the brain activity from fMRI signals without prior knowledge. Our findings show that the A4D-fMRI enabled us to solve an important problem, coupling the spatial and the temporal dimension and to recover brain activations overlapping the ones obtained with the GLM. Our results also show higher correlations of the recovered time courses with the ground truth compared to the TA. This opens a new channel for the analyses of rs-fMRI data and the recovery of paradigm-free neural activity to be used for investigations in future clinical applications.

## Data Availability Statement

Publicly available datasets were analyzed in this study. This data can be found at: https://db.humanconnectome.org/app/template/Login.vm;jsessionid=55C7D675B7D4CA21400384E01478FD3A.

## Author Contributions

IC, RD, and SD-G: study conception and design, data collection, analysis and interpretation of results, and manuscript preparation. All authors contributed to the article and approved the submitted version.

## Funding

This work has received funding from the European Research Council (ERC) under the European Union's Horizon 2020 research and innovation program (ERC Advanced Grant agreement No 694665: CoBCoM—Computational Brain Connectivity Mapping).

## Conflict of Interest

The authors declare that the research was conducted in the absence of any commercial or financial relationships that could be construed as a potential conflict of interest.

## Publisher's Note

All claims expressed in this article are solely those of the authors and do not necessarily represent those of their affiliated organizations, or those of the publisher, the editors and the reviewers. Any product that may be evaluated in this article, or claim that may be made by its manufacturer, is not guaranteed or endorsed by the publisher.

## Footnotes

^1^ gitlab.inria.fr/cobcom/a4dfmri

^2^ https://fsl.fmrib.ox.ac.uk/fsl/fslwiki

^3^ https://fsl.fmrib.ox.ac.uk/fsl/fslwiki/POSSUM

^4^ https://miplab.epfl.ch/index.php/software/total-activation
