# Supplementary material for: An Anisotropic 4D Filtering Approach to Recover Brain Activation From Paradigm-Free Functional MRI Data
Source: Front Neuroimaging. 2022 Apr 1;1:815423. doi: 10.3389/fnimg.2022.815423 (PMC10406250; doi:10.3389/fnimg.2022.815423)
Supplement: Supplementary file 1 [file Data_Sheet_1.pdf]

# Supplementary Material

## Figures

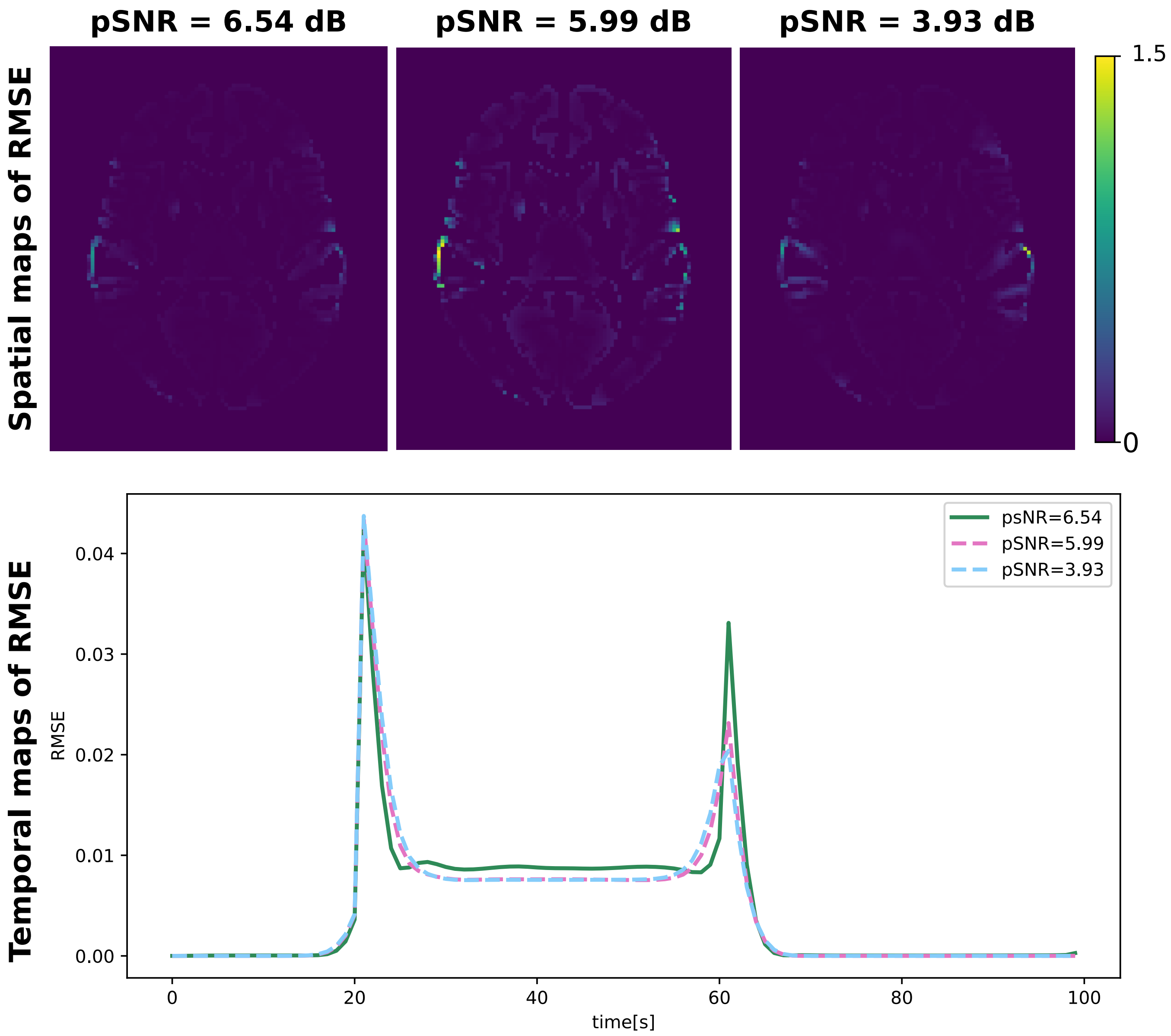


**Figure S1.** Results are obtained from synthetic data. A 3D activation map was multiplied by a piece-wise constant signal of 100 s, with one onset of 40 s, from 20 s to 60 s, and corrupted by model and additive noise. The performance of the A4D-fMRI was tested on several simulated 4D fMRI images obtained by adding different amount of noise for each experiment. The whole image was regularized using the A4D-fMRI and the voxel-wise activity-inducing signals were recovered.
From top to bottom: spatial maps of root mean square error (RMSE) computed among all the time points et each separate voxel. Each colums corresponds to a different peak-SNR (pSNR): 6.54 dB, 5.99 dB, 3.93 dB from left to right. At the bottom: temporal maps of RMSE computed among all voxels at each time point. Results show and confirm that we do not perform well at edges because the recovered activation is smooth.

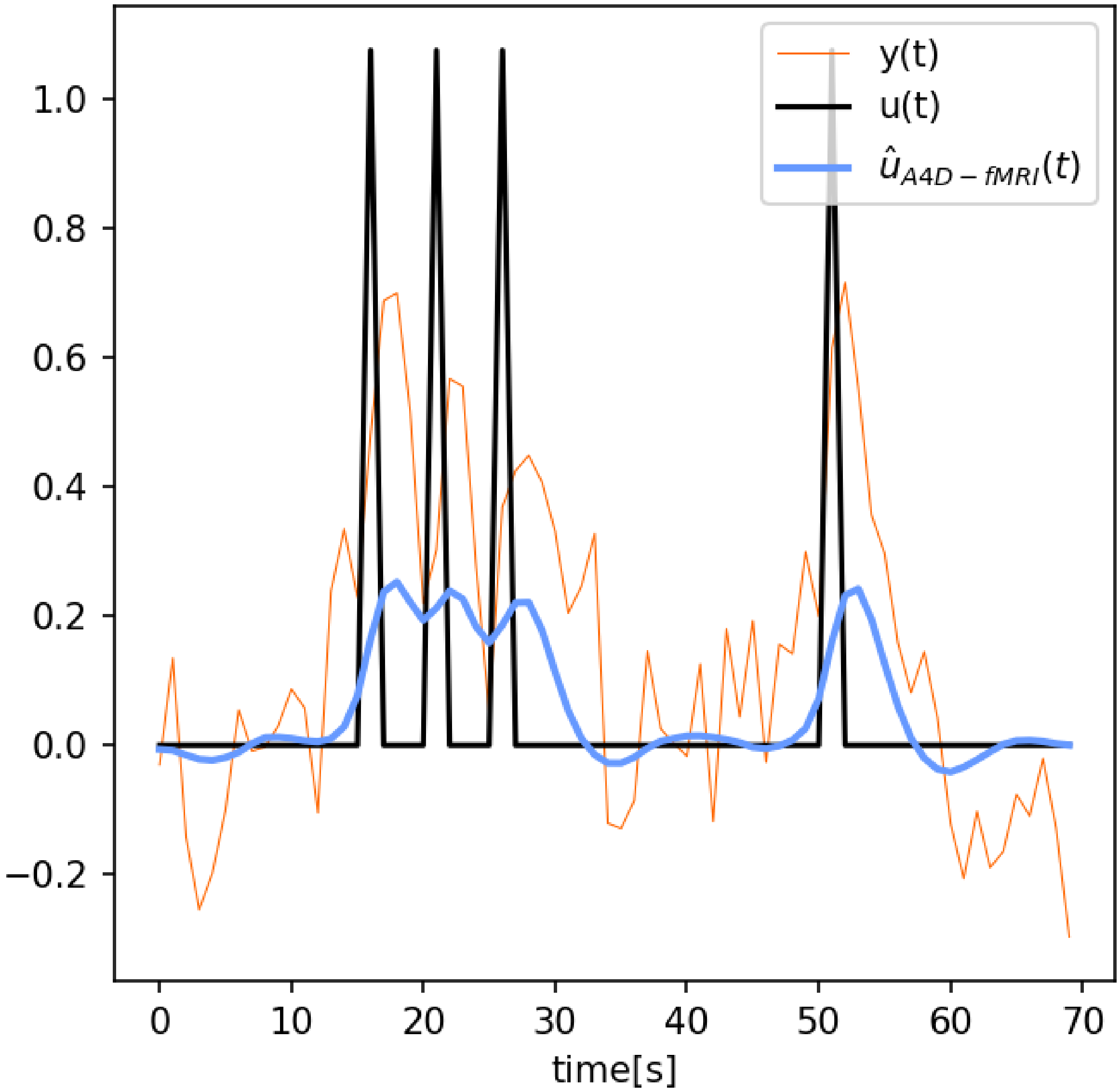


**Figure S2.** To investigate the performance of the A4D-fMRI on event-related designs, for each voxel of the 3D activation map, brain activations were simulated as spike train. The 4D datasets were corrupted with model and additive noise. To recover the voxel-wise activty inducing signals, the A4D-fMRI algorithm was applied on the noisy synthetic 4D fMRI images. The plot shows a reconstructed time series $\hat{u}(t)$ obtained with the A4D-fMRI ($\hat{u}_{A4D-fMRI}(t)$, light-blue) superimposed on the spike activation ($u(t)$, black) and fMRI signal ($y(t)$, orange). Peak-SNR = 12.41 dB.

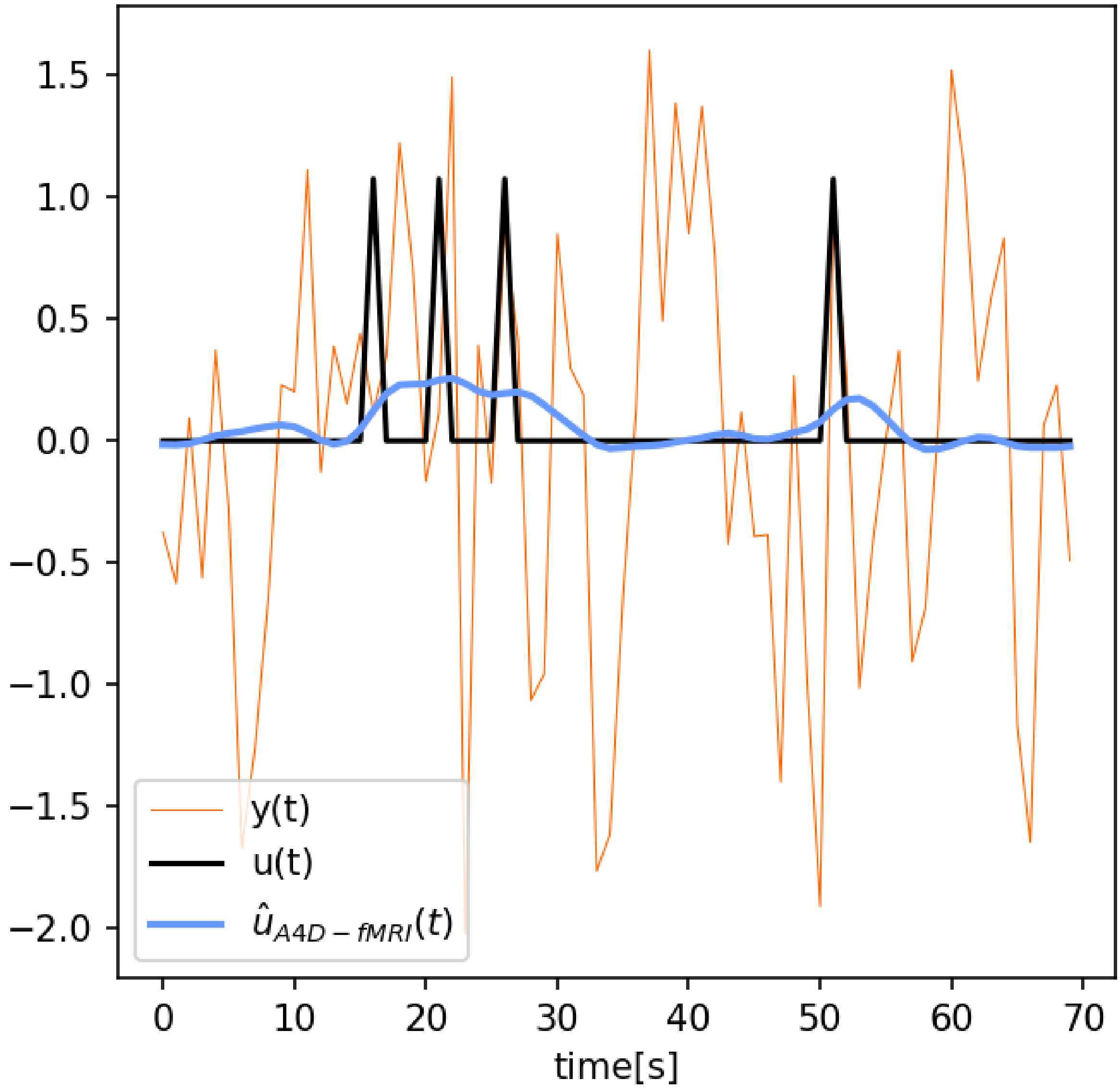


**Figure S3.** To investigate the performance of the A4D-fMRI on event-related designs, for each voxel of the 3D activation map, brain activations were simulated as spike train. The 4D datasets were corrupted with model and additive noise. To recover the voxel-wise activty inducing signals, the A4D-fMRI algorithm was applied on the noisy synthetic 4D fMRI images. The plot shows a reconstructed time series $\hat{u}(t)$ obtained with the Anisotropic 4D-fMRI (A4D-fMRI) ($\hat{u}_{A4D-fMRI}(t)$, light-blue) superimposed on the spiky activation ($u(t)$, black) and fMRI signal ($y(t)$, orange). Peak-SNR = 8.51 dB.

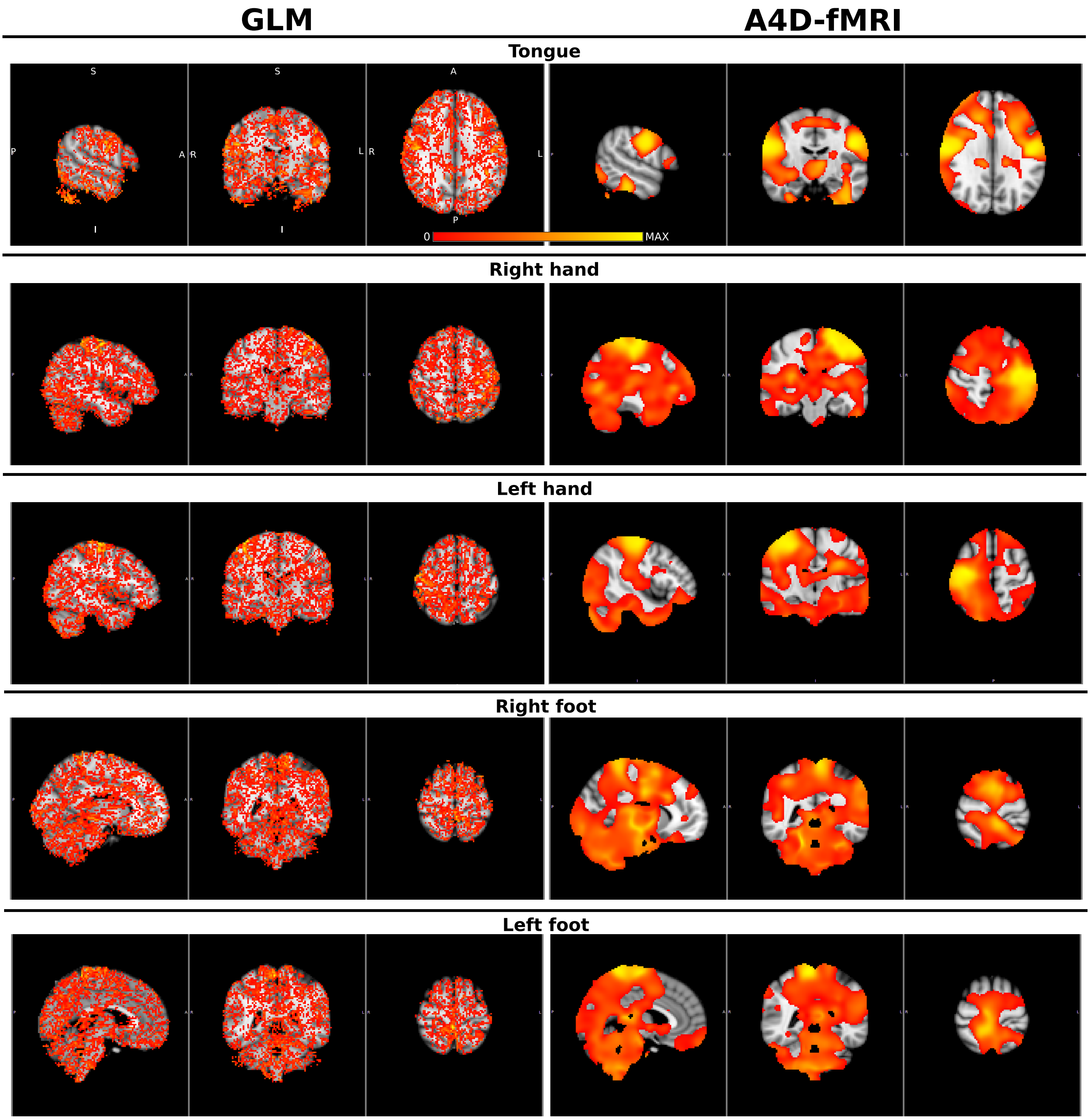


**Figure S4.** Qualitative comparison between the GLM and the Anisotropic 4D-fMRI (A4D-fMRI) approach. On the left column, superimposed to the standard MNI template, the $\beta$-regressors map obtained using the GLM implemented in FSL. On the right column, the whole-brain voxel-wise correlation maps obtained using the A4D-fMRI superimposed to the standard MNI brain. The Pearson correlation $(r)$ was computed voxel-wise across the whole brain, between the reconstructed activity inducing signals $\hat{u}(t)$ and the five motor tasks simulated as piece-wise constant signals with ones in the time points where the subject is executing the task and zeros elsewhere. Each row corresponds to a specific motor task, from the top to the bottom: the tongue, the right and left hand, and the right and left foot. The image does now not have a lower threshold. For a correct visualization of results we only visualize positive values. Nevertheless, the maximum values are not the same ones for the beta values that can be greater than 1 (and -1) and the correlation values that instead are between -1 and 1. Because of this, for each line in the figure, the colorbar MAX corresponds to the maximum value of the $\beta$-regressors (for GLM) and of the r-values (for the A4D-fMRI). A: anterior; P: posterior; S:superior; I: inferior; R: right; L: left.

## Supplementary Discussion

In this paragraph we will provide further discussion regarding the merits of having a good estimate in terms of signal amplitude.

If we are looking for transients, as in the iCAPs framework (Karahanoğlu and Van De Ville, 2015: `https://doi.org/10.1038/ncomms8751`), amplitude is not as essential to recover, but instead very sharp transients are crucial. Nevertheless, if we aim at performing connectivity analysis, for example in structure–function mapping (Deslauriers-Gauthier et al., 2020: `https://doi.org/10.1016/j.media.2020.101799`), recovering the correct value (or at least relative value) for the amplitude is important. Furthermore, Figures 4 (simulations) and 9 (task data) show an improved correlation between the recovered signal and the stimuli when using A4D-fMRI. As this measure is invariant to amplitude differences, it clearly highlights that A4D-fMRI provides a improved reconstruction of the shape of the underlying activation.
